# Books

**Published:** 1882-12

**Authors:** 


### Books.

---

Slight Ailments: Their Nature and Treatment. By Lionel S. Beale, M.B., F.R.S. Second edition, enlarged and illustrated. Philadelphia: P. Blakiston, Son & Co. 1882. Pp. 283. Cloth. Price, $1 25.

This new and revised edition of Dr. Beale's work is published simultaneously with the London edition by special arrangement with the author; and it constitutes one of a large series of useful and inexpensive books which this well-known publishing house offers to the medical profession.

The volume before us is devoted to such topics as the tongue in health and disease, the appetite, nausea, thirst, hunger, indigestion, constipation, diarrhœa, worms, vertigo, biliousness, sick headache, neuralgia, rheumatism, etc., a class of commonplace and constantly recurring disorders that make up a large part of the business of the general practitioner, and which are usually only referred to casually or incidentally in the ordinary text-books.

Appropos of the prevailing—not to say fanciful—pathological theories of the day, we cannot resist the temptation to transcribe a common sense paragraph from the chapter on the tongue. It reads: * * * * *

"I have seen every part of the cavity of the stomach, and the small and large intestines of an infant filled with curdled milk which had not undergone the slightest digestion, and every particle of which, when under the microscope, seemed to be almost composed of bacteria, so abundant were these bodies. Sometimes, however, bacteria grow and multiply in the milk of the mother before it has escaped from the breast, and the changes effected in the milk by the growth and multiplication of the organisms, it need scarcely be said, render it quite unfit for the sustenance of the infant; and such milk, were it taken, would, except perhaps in the very strongest children, give rise to serious derangement of the digestive organs. In such a case the maternal secretion must have been out of order at the time of its secretion, or the bacteria would not have grown and multiplied in it. It is certain that in such secretions and the glands which produce them, bacteria-germs are always present, but do not increase and multiply until long after the secretion has been discharged from the gland.

"In the face of such facts, it is difficult to accept the doctrine that bacteria, fungi, and such like low organisms, are morbific, or disease-producing agents, or are of themselves productive of harm to the organism into which they pass. It is astonishing that, notwithstanding these facts, which can be verified by any one—facts with which many of us have been familiar for the last thirty years or more—should at this time be unknown to, or somehow escape the cognizance of, some who have been recently studying the life-history of these very organisms. The knowledge of such broad and general facts renders it difficult, I think, to accept off hand the doctrine that such organisms are somehow intimately connected with the origin and communication of many of the most serious diseases of man and animals. Of late years, however, the theory that such organisms, which are invariably present in all decaying healthy normal structure, or closely allied organisms, or their pathologically, modified descendants, constitute the actual poison of most of the contagious diseases which invade us, has spread far and wide, and has been accepted by many as a general principle."

Notwithstanding the apparent paucity of the therapeutical resources of the writer, the volume is one that we can commend as containing many practical and useful suggestions.

---

A Practical Laboratory Course in Medical Chemistry. By John C. Draper, M.D., LL,D. New York: William Wood & Co. 1882.

"It is the object of the following course," says the author, "to give to medical students sufficient practice in chemical manipulations to enable them to perform in a satisfactory and reliable manner those tests which are required of the practicing physician, and also to give them some experience in the use of chemical symbols, formulæ and equations."

In printing the book every other page has been left blank in order that the student may be enabled conveniently to record, in its proper place, the results of the experiments he makes, and of additional facts obtained from oral instruction.

Professor Draper has prepared a book which a casual examination convinces us will prove indispensable to the ambitious and intelligent student and of great service also to the medical practitioner, in conducting those tests and chemical manipulations inseparable from the skillful and exact practice of our art.

The volume is small and inexpensive, yet sufficiently comprehensive for the ordinary uses of medical men and to meet the ordinary requirements of the student. It is a handy little assistant that will win its way along the ranks of progressive physicians and earnest students of medicine, whether in college or out of it.

---

A Guide to Therapeutics and Materia Medica. By Robert Farquharson, M.D., Edinburg, F.R.C.P., London. Third American Edition Revised by the Author. Enlarged and Adapted to the U. S. Pharmacopœia, By Frank Woodbury, M.D. Philadelphia: Henry C. Lea's Son & Co. 1882. Pp. 526.

"The present volume," says the editor, "is an intelligent effort to present, in moderate compass, such well-digested facts concerning the physiological and therapeutical action of remedies as are reasonably established up to the present time. By a convenient arrangement the corresponding effects of each article in health and disease are presented in parallel columns, not only rendering reference easier, but also impressing the facts more strongly upon the mind of the reader."

The constantly increasing number of remedies and the many really useful drugs that year by year are added to our materia medica renders a knowledge of these advances essential to the active practitioner. This work seeks to supply this need. It does not differ materially from others of its kind except in the double column arrangement referred to in the extract from the editor's preface.

The introductory chapter relates chiefly to the methods of administering remedies, and rules for prescribing. At the end of the volume is a ready reference table of poisons and their antidotes, also tables of weights and measures.

The typographical peculiarities and an excellent index, both of subjects and also of the diseases referred to in the text, enhances the value of the book for reference.

We cannot, however, close this notice without directing attention to what appears to us to be a dangerous suggestion in regard to the administration of belladonna to children, whom the author asserts exhibit a remarkable insusceptibility to its effects. On page 45 we read "the dose of which [the tincture] I have pushed, in a child of ten years of age suffering from incontinence of urine, to ʒij with good effect, and the development of only very mild forms of physiological disturbance. I commonly begin with twenty minims in a child of two or three, and I have prescribed ten minims in an infant of six months with remarkable benefit."

Notwithstanding we are warned in a foot note that this is the tincture of the British pharmacopœia, and is consequently two and a half times weaker than the American tincture, we are constrained to regard the dose as hazardous, and should consider it unsafe even if it were ten times weaker. Many persons are very susceptible to the influence of this agent and it should always be cautiously administered ; certainly until their idiosyncrasies in regard to it are ascertained.

**Fistula, Hemorrhoids, Painful Ulcer, Stricture, Prolapsus and Other Diseases of the Rectum:** Their Diagnosis and Treatment. By William Allingham, M.D. Fourth Revised and Enlarged Edition, with illustrations. Philadelphia: P. Blakiston, Son & Co. 1882. Pp. 168. Paper. Price 75 cents.

The reputation of the distinguished author as an authority upon the subjects discussed in this work is world-wide, and the popularity of this book is evidenced not alone by the exhaustion of three large editions, but by the further fact that it has been translated into the French, Italian, Spanish and Russian languages.

In the introductory chapter the author lays down the indisputable proposition that "An accurate diagnosis in rectal diseases is all-important, and to prescribe for patients suffering from these maladies without examining them, both ocularly and digitally, is not only false delicacy, but radically wrong, and likely to bring the treatment of these diseases into contempt."

Of fistula in ano he says: "I know many authorities have stated that it is only necessary to incise the fistula between its external and internal openings, and that the sinus above the internal opening will spontaneously close. My experience is most decidedly opposed to this statement. In the great majority of cases you will not cure your patient unless you lay the whole sinus open, from end to end. Over and over again I have left the sinus above the internal opening unintefered with, and almost invariably have had to regret having done so, and to perform a second operation." In the consideration of the propriety of operating for fistula upon phthisical patients, he sums up his opinion in these words: "For my own part, I do not think we have many, if any, clinical facts tending to show that the operation for fistula in phthisical patients renders the lung affection worse, or makes it more rapidly progressive. In saying this I must not be understood to advocate wholesale indiscriminate operations upon *tuberculous* patients; but I mean that if care be taken in the selection of proper cases, avoiding interference, if possible, with *rapidly advancing* phthisis, and the operation be performed discreetly, at the right time of the year, and with favorable surroundings, the patients will generally do well, and be benefitted and not damaged, by the cure of their rectal malady."

In the course of his discussion of the treatment of internal hemorrhoids, he says: "I have read, in American pamphlets, that the injection of carbolic acid into internal piles, for the purpose of effecting radical cures, is very commonly practiced in America, and that 'schools of quacks' perambulate the country armed with a hypodermic syringe and a bottle containing a so-called secret remedy, this remedy being carbolic acid diluted in different ways and of differing strength; the favorite formula is equal parts of strong carbolic acid, glycerine and water.

"I tried the injection plan on some few cases, but the result was much pain, more inflammation than was desirable, a lengthy treatment, and the result doubtful; certainly not a radical cure. It appears to me that all attempts to destroy vascular growths by causing coagulation of blood or inflammation in them, while they are not shut off from the general circulation, must be fraught with danger."

He indorses the ligature, of course, as *the* operation for the vast majority of cases of internal piles.

He thinks the only treatment for polypus of the rectum is removal, but adds the caution, "I do not think it safe either to cut or tear polypi off, as a troublesome arterial hemorrhage may ensue." A ligature should be applied to the pedicle before it is divided.

The style of the writer is clear and direct, and the work, as is well known, is worthy of unreserved confidence.

